# Daylight photodynamic therapy: patient willingness to undertake home treatment

**DOI:** 10.1111/bjd.17920

**Published:** 2019-06-05

**Authors:** L.J. McLellan, P. O'Mahoney, S. Logan, S. Yule, C. Goodman, A. Lesar, L. Fullerton, S. Ibbotson, E. Eadie

**Affiliations:** ^1^ School of Medicine University of Dundee Dundee U.K.; ^2^ The Scottish Photodynamic Therapy Centre NHS Tayside Ninewells Hospital Dundee U.K.; ^3^ Photobiology Unit NHS Tayside Ninewells Hospital Dundee U.K.

dear editor, In the U.K., almost one in four individuals over 60 years are affected by actinic keratoses (AKs),^1^ and this is a cause of significant morbidity in an ageing population, with risk of progression to squamous cell carcinoma.^2^ Daylight photodynamic therapy (dPDT) is an effective and simple treatment for field change AK, with similar efficacy to conventional PDT.^3^ Commonly, skin surface preparation is performed in a dermatology clinic prior to dPDT. However, a recent German study by Karrer and colleagues investigated dPDT as a patient‐applied home‐delivered treatment for face and scalp AK and reported that patients who undertook this self‐administered treatment had high levels of efficacy, tolerance and patient satisfaction.^4^

We surveyed patients who had previously received dPDT at Ninewells Hospital, Dundee to ascertain their experience of treatment, whether they would have liked dPDT sooner, whether they would consider doing the treatment themselves and what type of support they would require (https://www.researchgate.net/project/Smart-PDT). A questionnaire was sent to 56 patients and 35 were returned. An engagement event was also held, inviting nine survey participants of differing viewpoints (five attended) and six members of staff to discuss the questionnaire further and investigate potential service improvements (focusing on improving the current service and the potential for home treatment). Formal advice on the construction and content of the questionnaire, and on the organization, content and focus of the engagement event was provided by the National Health Service (NHS) Tayside improvement team, which is part of the Academic Health Science Partnership in Tayside. Approval of the use of the questionnaire and engagement event was obtained through the NHS Tayside local clinical governance process.

Consistent with previous published studies,^5^ most respondents experienced no problems during dPDT (63%) and 90% felt that clearance rates were the same as (32%) or better than (58%) other treatments that they previously underwent for AK. In this secondary care setting, most of our respondents had previously received several types of treatment prior to dPDT (61% 5‐fluorouracil, 52% diclofenac, 51% surgery, 45% cryotherapy, 36% imiquimod). A total of 54% of patients reported that dPDT was better tolerated and 27% stated similar tolerance to other AK treatments; although poor weather had caused difficulties for some (low temperature – six responses and rain – five responses). Respondents ranked minimal pain/discomfort, disease clearance, convenience of outdoor treatment and good cosmetic outcome as important factors for dPDT, with 82% being happy or very happy with the service.

It is apparent from our results and published studies that dPDT is a preferred and well‐tolerated treatment for AKs.^3^ It has also been reported that there is poor adherence with other forms of AK treatment.^1^ Currently, dPDT is only available in a limited number of locations in the U.K. and often these are secondary healthcare providers. Our respondents would have appreciated the opportunity to have treatment sooner in a non‐hospital setting (73%), with 12% preferring dPDT via general practitioner surgeries, 15% could be persuaded to undertake self‐treatment, 34% said they would be happy to self‐treat if adequate support was available and 12% would like to control and have some ownership of their own treatment.

Respondents were also asked their opinion on using a smartphone application to perform treatment at home and the support they would require using such an application. In total, 50% of respondents said they owned a smartphone or tablet and in order to use the application to perform dPDT at home 78% of respondents would want contact details of a dPDT nurse, while 61% wanted weather predictions and 61% required a step‐by‐step written guide. Half of respondents wanted the ability to send secure messages to a doctor or nurse, 44% wanted instructional videos (i.e. prodrug application and aftercare) and the same number required the ability to send pictures to the doctors or nurses. Only 11% would want audio instructions, with 17% selecting ‘other’ and highlighting the desire for accurate dosage reports throughout treatment. From the engagement event it was further clarified that it would be essential for the app to be intuitive and easy to use. The clear message from this event was that patients wanted choice – to have local treatment available or for self‐delivery at home.

To our knowledge, it has never previously been demonstrated that there is a willingness within the AK patient population to undertake dPDT treatment at home and to use smart technology to assist in such an endeavour. Our findings highlight the importance to patients of the availability of well‐tolerated, effective, convenient treatment for AKs, as well as the high value of including patient opinion in clinical service development.
